# Glandular odontogenic cyst associated with ameloblastoma: Case report and review of the literature

**DOI:** 10.4317/jced.53775

**Published:** 2017-06-01

**Authors:** Timothée Cousin, Samuel Bobek, Dolphine Oda

**Affiliations:** 1 DDS candidate. University of Washington School of Dentistry, Seattle WA USA; 2 MD, DMD. Swedish Hospital Maxillofacial Surgery, Seattle WA USA; 3 BDS, MSc. Department of Oral and Maxillofacial Surgery, University of Washington School of Dentistry, Seattle WA USA

## Abstract

Glandular odontogenic cyst (GOC) associated with ameloblastoma is an exceedingly rare histologic presentation with no known clinical significance or treatment applications. Four cases have been reported, three in the mandible and one in the maxilla. The age range is 14-65 and with male predilection. All four presented with swellings and two with pain. We add one more case to the literature of a 58-year old male presenting with an expansile multilocular radiolucency between teeth #19-23. The ameloblastomatous changes in this case are consistent with those of a unicystic ameloblastoma-mural subtype. Although the histologic changes are those of a unicystic ameloblastoma, the clinical and radiographic findings are not. This case therefore presents a clinical challenge with regards to treatment planning for recurrence and prognosis. We conclude that treatment for GOC therefore be based on individual clinical presentation.

** Key words:**Glandular odontogenic cyst, GOC, ameloblastoma, unicystic ameloblastoma, mural unicystic ameloblastoma.

## Introduction

Glandular odontogenic cyst (GOC) is an uncommon odontogenic cyst with an incidence of 0.12-0.2% of odontogenic cysts (1-3). The histological criteria for GOC have evolved since it was first described by Gardner *et al.* in 1988 and its diagnosis is less of a challenge today in light of an increasing number of reports and clearer definition of its diagnostic criteria (2,4,5). GOC has been traditionally described as predominantly affecting middle-aged individuals in their 4th-7th decades of life (mean=45.9 years-old) (2,3). While some studies report no significant gender predilection (1,3,6–8), others have reported a slight male predominance of 1.3:1 to 2:1 (2,9,10). GOC commonly affects the mandible 2-3x as often as it does the maxilla (1,2,7,8,10). Some reports claim a preference for the anterior mandible (3,6,8), but others indicate equal distribution between anterior and posterior mandibular segments (2). The radiographic findings of GOC often resemble those of odontogenic keratocyst or ameloblastoma, the former presenting as a unilocular radiolucency with scalloped borders and the latter as a multilocular and expansile radiolucency (1). Histologically, GOC may have features overlapping with botryoid odontogenic cyst, dentigerous cyst, and low-grade mucoepidermoid carcinoma (2,8), but not with ameloblastoma.

Ameloblastoma are described as the most frequently diagnosed odontogenic tumor (11) and often arise with clinical and radio-graphic features reminiscent of odontogenic cysts, including dentigerous cyst (12). Unicystic ameloblastoma (UA) is a frequent variant of ameloblastoma arising in a cystic structure. It is most commonly diagnosed in the second decade of life and has a strong predilection for the mandible (12). Unicystic ameloblastoma commonly presents as a unilocular radiolucency with corticated border with 50-80% associated with impacted third molars, closely resembling dentigerous cyst (12).

The connection between unicystic ameloblastoma and GOC has not been well established given the rarity of the condition. So far, only four cases of GOC are reported to be associated with ameloblastoma (4,13-15). None of the four reports have clearly defined the type of ameloblastoma present within the GOC lesion, but at least three point to unicystic ameloblastoma (4,13,14). In this manuscript, we present a case of GOC associated with clear ameloblastomatous changes consistent with those of the unicystic ameloblastoma-mural histologic subtype. We do not however have enough information to determine clinical behavior, treatment, or prognosis.

## Case Report

-Clinical Findings

**Figure 1:**
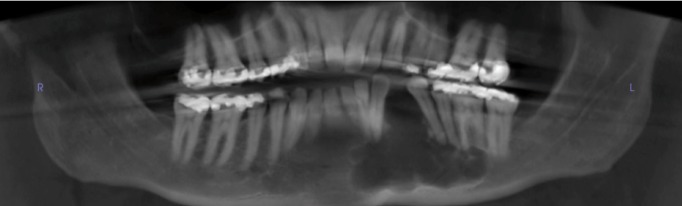
Panoramic radiograph at first presentation. This panoramic radiograph was taken at first presentation and displays a well-demarcated, multilocular radiolucency with scalloped border between teeth #19-23. The teeth are pushed apart, especially area between #21 and 22.

**Figure 2:**
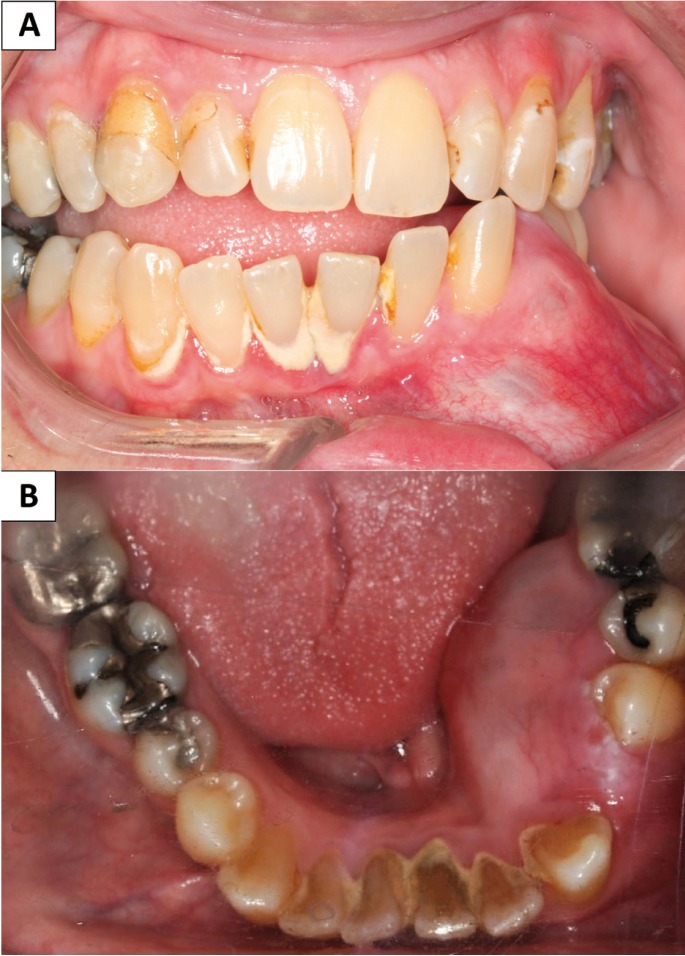
Clinical photographs at first presentation. A) Intraoral frontal view demonstrating a swelling in the left buccal posterior mandible expanding superiorly. Note the pale grey to light-blue color of the stretched alveolar mucosa. B) Occlusal view displaying lingual expansion into the floor of the mouth. The teeth have been displaced and pushed apart, especially #21 and 22.

A 58-year-old male presented with a slowly expansile lesion in the left posterior mandible which had been present for an unknown period. The teeth were vital and pushed apart, especially in the area of #21 and 22 (Fig. 1). The swelling was clinically obvious, protruding lingually into the floor of mouth and expanding buccally into the mandibular vestibule (Fig. 2). The swelling reflected buccally as translucent grey-blue through the mucosa (Fig. 2). The patient denied pain or paresthesia.

Radiographic Findings

Radiographically, a large, multilocular radiolucency with scalloped borders was present between teeth #19-23. The margins are clearly defined. Teeth #21 and #22 are clearly pushed apart. Tooth resorption was not identified and there was no evidence of alveolar bone perforation (Fig. 1).

Histological Findings

**Figure 3:**
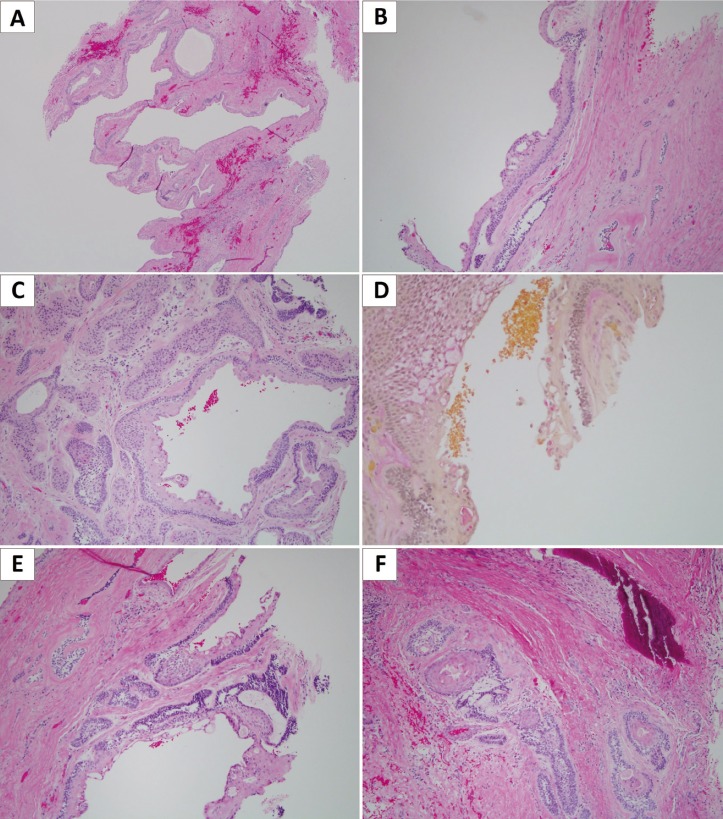
Histologic presentation of the GOC & Ameloblastoma. A) Cystic structure lined by epithelium exhibiting multifocal early ameloblastomatous changes among the typical histologic features of GOC (H&E stain: magnification 40x). B) Typical histologic features of GOC manifested in spaces within the lining epithelium lined by cuboidal epithelial cells (H&E stain: magnification 100x). C) Cystic lining epithelium with the superficial layer covered by cuboidal cells with “hobnail” appearance. One epithelial sphere can be observed. The connective tissue wall contains odontogenic neoplastic epithelial islands (H&E stain: magnification 100x). D) Sparse mucous producing cells in the lining epithelium (Mucicarmine stain: magnification 100x). E) Histologic features of ameloblastomatous changes in the GOC lining epithelium manifested by a layer of palisaded basal cells with hyperchromatic nuclei and focal reverse polarization. The basal layer is covered by stellate-reticulum type of epithelial cells (H&E stain: magnification 100x). F) Neoplastic odontogenic epithelial islands within the connective tissue wall. These islands are of variable shapes and sizes and are lined by one layer of palisaded and polarized cuboidal/columnar cells. The center of the islands contains squamous and stellate-reticulum type of epithelial cells (H&E stain: magnification 100x).

The hematoxylin and eosin (H&E) stained sections of an incisional biopsy showed a combined cystic and solid neoplastic process. The cystic structure had features of GOC in that the epithelium was of variable thickness (Fig. 3A), had glandular-like spaces within the lining epithelium (Fig. 3B), scant mucous producing cells which were positive with mucicarmine stain (Fig. 3D), and cuboidal epithelial cells with hobnail appearance layering the very superficial layer of the lining epithelium (Fig. 3C). In rare areas, epithelial spheres were noted (Fig. 3C).

The second component was the neoplastic ameloblastomatous changes which manifested in two forms (Fig. 3E,F). First, there were multifocal ameloblastomatous changes involving the deep layers of the lining epithelium including the basal cell layer (Fig. 3E). The latter was cuboidal, palisaded with focal reverse polarization. The basal cell layer was covered by stellate-reticulum type of epithelial cells. The connective tissue wall contained odontogenic epithelial neoplastic islands of variable shapes and sizes (Fig. 3F). The epithelial islands had follicular, acanthomatous and combined histologic morphology. The periphery of the islands was lined by one layer of palisaded cuboidal/columnar cells with reverse polarization typical of ameloblastoma histologic features.

## Discussion

The association of ameloblastoma with GOC is exceedingly rare. Only four such cases exist in the literature making the current case only the fifth to be reported. A review of the literature is presented in  Table 1, summarizing the clinical and radiographic pre-sentation of all five cases (4,13-15).

**Table 1:**
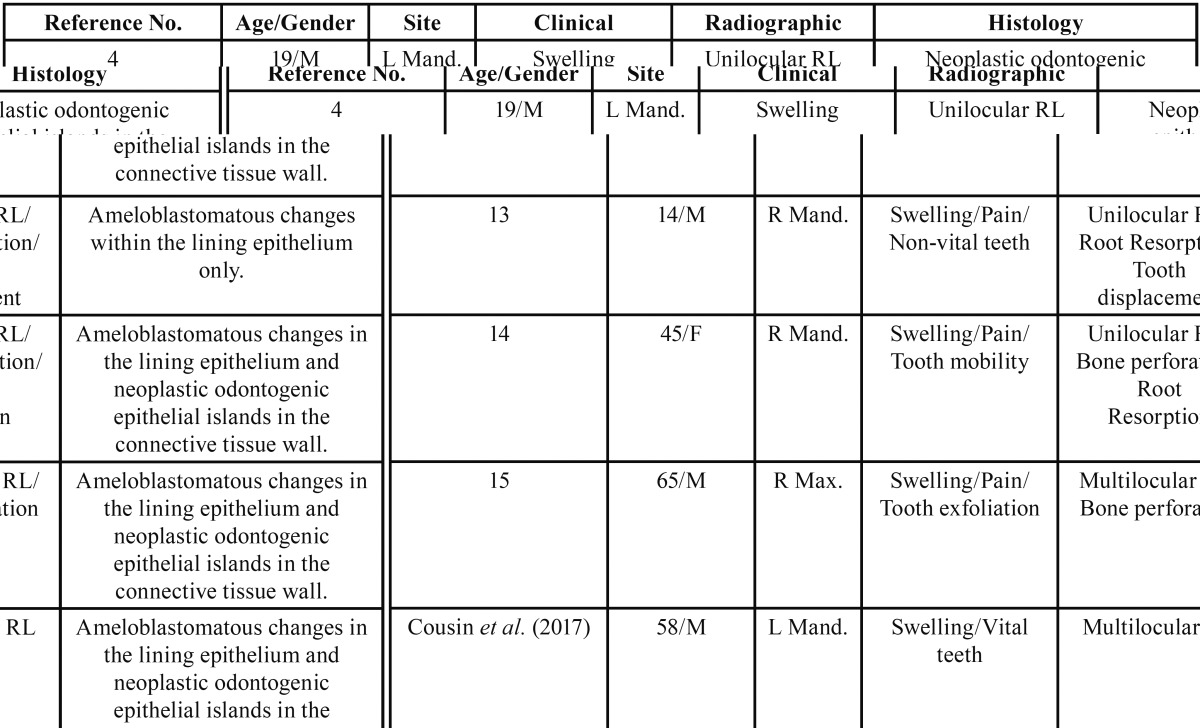
Review of all cases having received a final diagnosis of GOC associated with ameloblastoma in the literature.

Clinically, three of the four cases occurred in the posterior mandible extending anteriorly (4,13,14), and one occurred in the posterior maxilla, again extending anteriorly (15). The current case was also in the mandible between teeth #19-23, resulting in a mandible to maxilla ratio of 4:1. Of those cases occurring in the mandible, all three affected the right side (4,13,14). but one originated from tooth #19 (left mandible) and extended to tooth #27 (4). The current case was on the left side of the jaw. In the maxilla, the reported case was located between the left central incisor to the right first molar (15). All four cases and the current case presented with extensive swelling and expansion, and three cases presented with pain (13-15). The teeth in the current case were all vital, a characteristic observed in only one of the four cases previously reported (14). The age range for the four reported cases is 14-65 (mean=36-years-old) with a male to female ratio of 3:1 (4,13-15). The present case is that of a 58-year-old male, older than the mean age, but within the age range described in the literature.

Radiographically, all four cases including the present one were radiolucent and expansile. Three of the previously reported cases were unilocular (4,13,14), and one was multilocular (15). The current case was also multilocular. Root resorption was described in two of the four cases (13,14); the current case did not show root resorption. Three cases demonstrated displacement of teeth and so did the current case (13-15). Finally, two cases were described to perforate either or both the buccal or lingual plate (14,15). The current case did not perforate bone.

Histologically, the current case demonstrates clear change from cyst to neoplasm within the lining epithelium (Fig. 3); such clear change is also noted in three of the reported cases (13-15). Gardner only described odontogenic epithelial islands in the wall of cyst (4). The current case shows the presence of odontogenic epithelial islands in the wall of the cysts, a feature shared by three of the previously reported cases (4,14,15), with the exception of Kumaraswamy who observed ameloblastomatous changes arising only in the lining epithelium (13). Thus far, two of the reported cases as well as the current case show ameloblastomatous changes arising in the lining epithelium with a solid epithelial island component in the connective tissue wall (14,15). These features are consistent with those of unicystic ameloblastoma-mural subtype and widely described to occur in dentigerous cysts (12).

None of the four reported cases commented on the clinical behavior of such histologic changes within GOC, neither did these reports address prognosis and recurrence rate. It is too soon for treatment, prognosis, and recurrence to be predicted on such a small sample. As such, data from ameloblastoma arising in dentigerous cyst should not be extrapolated to GOC associated ameloblastoma. These two cysts differ widely in their clinical, radiographic, or histologic presentations, and even more certainly in their behavior, prognosis, and recurrence rate.

In conclusion, we present a case of ameloblastoma associated with GOC in a 58-year-old male who presented with extensive expansion of the mandible with a multilocular radiolucent radiographic appearance.

## References

[1] Magnusson B, Göransson L, Odesjö B, Gröndahl K, Hirsch JM (1997). Glandular odontogenic cyst. Report of seven cases. Dentomaxillofac Radiol.

[2] Kaplan I, Anavi Y, Hirshberg A (2008). Glandular odontogenic cyst: a challenge in diagnosis and treatment. Oral Dis.

[3] Faisal M, Ahmad SA, Ansari U (2015). Glandular odontogenic cyst – Literature review and report of a paediatric case. Journal of Oral Biology and Craniofacial Research.

[4] Gardner DG, Kessler HP, Morency R, Schaffner DL (1988). The glandular odontogenic cyst: an apparent entity. J Oral Pathol.

[5] Kramer IR, Pindborg JJ, Shear M (1992). The WHO Histological Typing of Odontogenic Tumours. A commentary on the Second Edition. Cancer.

[6] Hussain K, Edmondson HD, Browne RM (1995). Glandular odontogenic cysts: diagnosis and treatment. Oral Surgery, Oral Medicine, Oral Pathology, Oral Radiology, and Endodontology.

[7] Noffke C, Raubenheimer EJ (2002). The glandular odontogenic cyst: clinical and radiological features; review of the literature and report of nine cases. Dentomaxillofacial Radiology.

[8] Fowler CB, Brannon RB, Kessler HP, Castle JT, Kahn MA (2011). Glandular odontogenic cyst: analysis of 46 cases with special emphasis on microscopic criteria for diagnosis. Head Neck Pathol.

[9] Shen J, Fan M, Chen X, Wang S, Wang L, Li Y (2006). Glandular odontogenic cyst in China: report of 12 cases and immunohistochemical study. J Oral Pathol Med.

[10] Macdonald-Jankowski DS (2010). Glandular odontogenic cyst: systematic review. Dentomaxillofac Radiol.

[11] Johnson NR, Gannon OM, Savage NW, Batstone MD (2014). Frequency of odontogenic cysts and tumors: a systematic review. J Investig Clin Dent.

[12] Philipsen HP, Reichart PA (1998). Unicystic ameloblastoma. A review of 193 cases from the literature. Oral Oncol.

[13] Kumaraswamy Naik LR, Agarwal R, Amberkar VS, Ahmed Mujib BR (2008). A rare variant of ameloblastoma associated with a glandular odontogenic cyst. Indian J Pathol Microbiol.

[14] Hisatomi M, Asaumi J, Konouchi H, Yanagi Y, Kishi K (2000). A case of glandular odontogenic cyst associated with ameloblastoma: correlation of diagnostic imaging with histopathological features. Dentomaxillofac Radiol.

[15] Ponniah I, Murali Gopika Manoharan GV, SureshKumar P, Karthikeyan K (2007). How to name it: a rare case of odontogenic cyst. J Oral Pathol Med.

